# Effect of New Antidiabetics on Steatosis in Nerve Tissues and Nerve Conduction Velocity: Possible Role of Nerve Growth Factor (NGF)/Synaptophysin and Nrf2/HO-1 Pathways

**DOI:** 10.7759/cureus.65726

**Published:** 2024-07-30

**Authors:** Nehal H.M. Abdel-Halim, Elsayed A Eid, Yomna M Yehya, Medhat Taha, Ahmed A.H. Mosa, Omar Ammar, Ahmed N.A. Nasr, Emadeldeen Hussin, Abdelaziz M Hussein

**Affiliations:** 1 Department of Medical Physiology, Faculty of Medicine, Mansoura University, Mansoura, EGY; 2 Department of Internal Medicine, Faculty of Medicine, Delta University for Science and Technology, Gamasa, EGY; 3 Department of Anatomy, Umm Al-Qura University, Al-Qunfudhah, SAU; 4 Department of Anatomy and Embryology, Faculty of Medicine, Mansoura University, Mansoura, EGY; 5 Department of Neurology, Faculty of Medicine, Delta University for Science and Technology, Gamasa, EGY; 6 Department of Basic Sciences, Delta University for Science and Technology, Gamasa, EGY

**Keywords:** semaglutide, dapa, ngf/synaptophysin, nrf2/ho-1, obese, nerve conduction velocity

## Abstract

Objectives: The current study aims to investigate the impact of the GLP1 analog (semaglutide) and SGLT2 inhibitor (dapagliflozin) on nerve functions, morphology, and the underlying mechanisms involving nerve growth factor (NGF)/synaptophysin and Nrf2/HO-1 pathways in obese rats.

Methods: Forty male Sprague Dawley rats, aged six to eight weeks, were classified into five groups; normal group (high-fat diet {HFD} for 12 weeks, metformin group (HFD for 12 weeks + metformin in last four weeks), dapagliflozin group (HFD for 12 weeks +dapagliflozin in last four weeks, semaglutide group (HFD for 12 weeks + semaglutide in last four weeks). At the end of the experiment, the sciatic nerve was collected for nerve conduction study, oxidative stress marker (malondialdehyde, i.e., MDA), real-time polymerase chain reaction (PCR) study (for HO-1 and Nrf2), oil red O staining, electron microscopic examination and immunohistochemistry for NGF and synaptophysin.

Results: The HFD group showed a significant rise in blood glucose, serum lipids, homeostatic model assessment (HOMA) index, lipid deposition in nerve tissues, and lipid peroxidation (MDA) in nerve tissues with significant attenuation in nerve conduction velocity (NCV), the expression of Nrf2 and HO-1 genes and significant attenuation in area stained with NGF and synaptophysin. On the other hand, pretreatment with either dapagliflozin or semaglutide led to considerable enhancement in the deteriorated serum and nerve tissue parameters and reversed the pathological changes.

Conclusion: New antidiabetic drugs like SGLT2 inhibitors (more powerful) and GLP1 analog might have neuroprotective beneficial effects besides controlling the glycemic state in obese rats. This effect may result from reduced oxidative stress and increased Nrf2 levels, HO-1, synaptophysin, and NGF in the nerve tissues of obese rats.

## Introduction

Obesity, defined as having a body mass index (BMI) of 30 kg/m2 or higher, is now one of the leading causes of avoidable death in the 21st century and is rapidly spreading worldwide. According to data from the WHO, the number of obese persons globally has doubled since 1980. Obesity causes an inordinate amount of ectopic fat to accumulate in the liver, pancreas, heart, kidneys, and skeletal muscles, which can have negative clinical and metabolic effects [1]. The development of a greater number of severe health issues, such as type 2 diabetes (T2DM), metabolic syndrome (MetS), and cardiovascular disease, as well as a strong correlation with increased mortality [2]. One of the primary underlying pathophysiological characteristics of metabolic disorders is chronic low-grade inflammation, which is frequently brought on by excessive abdominal fat accumulation and is linked to an at least two-fold increased chance of T2DM and MetS [3]. Additionally, the early start of T2DM and insulin resistance (IR), the two most significant problems linked to obesity, result in the earlier beginning of progressive neuropathy. Electrophysiological tests on obese patients with poor glucose tolerance have recently revealed peripheral nerve damage. The pathogenesis of peripheral neuropathy may be influenced by hyperglycemia, insulin resistance, and/or other obesity-related problems [4].

At the fusion complex alone, up to 30 proteins govern synaptic vesicle release; synaptophysin is the most functional pre-synaptic protein. Synaptophysin, a vesicular transmembrane protein suggested to work as a cholesterol-binding protein, forms ion channels through the phospholipid bilayer and a calcium sensor. In addition, it is important when combined with synaptobrevin (VAMP2), which is essential for vesicle fusion [5]. One of the most prevalent neurotrophic factors is called nerve growth factor (NGF) which is among the most common neurotrophic factors which belong to the neurotrophin (NT) family. It is stated that NGF exhibits neurite-stimulating activity. NGFs are important neurotrophic factors for the maintenance and development of the central and peripheral nervous system. NGF also plays a crucial role in the degeneration and healing processes associated with neurological diseases [6]. So, the expression of both synaptophysin and NGF can help us to assess nerve growth and repair in our model of neuropathy.

Several anti-hyperglycemic drugs such as metformin, liraglutide, pioglitazone, and dapagliflozin revealed beneficial effects in obesity. As far as we are aware, ectopic fat accumulation in the nerve tissues with assessment of nerve conduction velocity in dietary obesity while comparing the effect of different anti-hyperglycemic drugs, e.g., dapagliflozin (SGLT2 inhibitor) and long-acting GLP1 analog semaglutide. Therefore, the objective of this study is to compare the effects of new antihyperglycemic drugs (dapagliflozin and semaglutide) on fat accumulation in nerve tissues, nerve conduction velocity (NCV), and related molecular pathways (NGF/synaptophysin and Nrf2/HO-1) in high-fat diet (HFD)-induced obese rats.

## Materials and methods

Experimental animals

About 60 male Sprague Dawley rats, weighing 150-200 grams, and aged six to eight weeks were used in this study. The Medical Experimental Research Center (MERC), located at Mansoura University, Mansoura, Egypt, housed and bred animals. Our local ethics committee gave its approval to all experimental protocols (code # R.21.12.1551).

Experimental design

The rats were divided randomly into five groups (eight rats each).

**Table 1:** Experimental groups. HFD, high-fat diet.

Group 1	Normal group (12 rats)	Rats fed on an ordinary chow diet.
Group 2	High-fat diet (HFD) (12 rats)	Rats were fed HFD for 12 weeks.
Group 3	Metformin group (12 rats)	Rats were fed HFD for 12 weeks + metformin (200 mg/kg/day) dissolved in distilled water via gastric gavage in the last 4 weeks [7].
Group 4	Dapagliflozin group (12 rats)	Rats were fed HFD for 12 weeks + dapagliflozin (1 mg/kg dissolved in 0.5 mL saline via oral gastric gavage) and 0.5 mL via subcutaneous saline injection daily for 4 weeks [8].
Group 5	Semaglutide group (12 rats)	Rats were fed HFD for 12 weeks + semaglutide (once a week (0.42 mg/kg/w) injected subcutaneously in 0.5 ml saline [9].

Collection of blood samples and tissues

By the end of the experiment (at the end of the fourth week of treatment), the rats were euthanized using a high dose of Na-thiopental (120 mg/kg, intraperitoneal), and blood samples were collected via cardiac puncture. Blood samples were rapidly centrifuged and serum was stored at -20°C for further biochemical analyses. Then the sciatic nerve was dissected from the muscles of the posterior aspect of the hindlimb of the rat and harvested. The sciatic nerves from six rats in each group were rapidly placed in a nerve chamber for doing a nerve conduction study, while those obtained from the remaining rats in each were divided into three parts. One part was stored in liquid nitrogen for biochemical examination and polymerase chain reaction (PCR) study, the second part was placed in a frozen section for oil red O stain, and the third part was placed in formalin, then in paraffin block for histopathological and immunostaining examination.

Estimation of sciatic nerve electrophysiological parameters

A gluteal muscle incision had been used to present and subsequently remove the sciatic nerve. Two of the metal rungs on either side of the MLT012/B nerve bath or chamber were linked with the black and red alligator clips from the stimulator electrodes. The nerve conduction velocity calculation in the sciatic nerve was calculated using the PowerLab 30/4 system and LabChart 7.0 software from ADInstruments, Bella Vista, New South Wales, Australia according to previous studies in meters/secs [10].

Investigations

Assessment of Serum Insulin, Glucose, and HOMA-IR Index Calculation and Lipid Profile (Cholesterol, Triglycerides)

Serum levels of fasting glucose, cholesterol, and triglycerides were measured by a spectrophotometer (Robonik Prietest Touch Plus Biochemistry Analyzer, Robonik India Pvt. Limited, Ambernath, Maharastra, India) using commercially available kits (SPINREACT, Barcelona, Spain) according to the manufacturer's instructions. Additionally, serum fasting insulin was determined in accordance with manufacturer guidelines using enzyme-linked immunosorbent assay (ELISA) insulin kits for rats from Sun-Red Biology and Technology (Shanghai, China, Cat # 201-11-0708) by Robonik ELISA reader (India). Then, using fasting insulin and fasting blood glucose, the insulin homeostasis model assessment (HOMA)-insulin resistance (IR) index was determined.

Assay of Marker of Oxidative Stress (MDA Concentration) in Nerve Tissues

Using a mortar and pestle, 50-100 mg of nerve tissue was homogenized in 1-2 ml of cold buffer (50 m M potassium phosphate, pH 7.5, 1 m M ethylenediaminetetraacetic acid {EDTA}). The homogenate was then centrifuged for 15 minutes at 4°C at 4,000 rpm. The supernatant was kept at -20°C until oxidative stress biomarkers were analyzed. Malondialdehyde (MDA) concentration in the supernatant of the homogenates was evaluated using a colorimetric technique as directed by the manufacturer (Bio-Diagnostics, Dokki, Giza, Egypt, Cat # MD2529) using a spectrophotometer (Robonik Prietest Touch Plus Biochemistry Analyzer, India).

Histopathological Examination for Fat Contents in Frozen Nerve Tissues

Using standard techniques, a slice of the nerve tissues was taken out and weighed to create fresh frozen sections that had been stained with oil red O stain. Using Olympus CX51 (Olympus Corporation, Shinjuku City, Japan) microscopy, oil red O-stained slices were investigated for lipid accumulation inside the tissues. Image-Pro Plus software (Media Cybernetics, Rockville, MD, USA) was used to take photomicrographs and evaluate them using color cube-based selection criteria.

Histopathological Examination of the Nerve Morphology by Hematoxylin and Eosin (H&E)

A piece of nerve tissue was removed, fixed in neutral buffered formalin at a 10% concentration, embedded in paraffin, sectioned at a thickness of 3 μm, and stained with H&E. Using a light microscope (Leica DM500 with camera Leica ICC50HD) and camera software LEICA Application Suite (LAS) EZ, Version 3.1.1 (Leica Microsystems, Heerbrugg, Switzerland) at Physiology and Biotechnology Laboratory, Department of Animal Production, Faculty of Agriculture, Mansoura University, slides were examined for indications of damaged tissues, abnormal cells, unbalanced cytoplasmic diffusion, edema, Invasion of inflammatory cells, irregular nuclei, and rupture of cells.

Transmission Electron Microscope (TEM) Examination

The left sciatic nerves from each group (four from each group) were preserved in 4% glutaraldehyde, processed, and then examined for ultramicroscopic examination. Ultrathin slices were stained with uranyl acetate and lead citrate (70 nm), which were then scanned using a transmission electron microscope (TEM-1400, JEOL, Tokyo, Japan) at magnifications of 4,000, 8,000, and 12,000. According to axon diameter, fiber types were divided into three categories: small (5 μm), medium (5-10 μm), and big (>10 μm) [11]. Using ImageJ software (National Institutes of Health, Bethesda, MD, USA), we counted the number of myelinated axons, the proportion of aberrant fibers (fibers with atypical forms, infoldings, or compressed myelin), and the values of the g-ratio (the ratio of the inner axon diameter to the outer diameter of the myelin sheath, which was used to assess axonal myelination for each group of nerve fibers) [12,13].

RNA Extraction and Real-Time PCR for mRNA of Nrf2, HO-1 Genes in the Tissues

According to the manufacturer's recommendations (Invitrogen Corporation, Grand Island, New York, USA), 50-100 mg of each tissue were kept in liquid nitrogen and homogenized in 1 ml of Trizol to get total RNA. One µg of total RNA was used for reverse transcription, along with a cDNA kit (high-capacity cDNA archive kit). The primers for the genes under test were in the following order: Nrf2: forward: 5′-GCTATTTTCCATTCCCGAGTTAC-3′, reverse: 5′ATTGCTGTCCATCTCTGTCAG-3′ and; for HO-1: forward: 5’-ATGGCCTCCCTGTACCACATC-3’, reverse: 5’-TGTTGCGCTCAATCTCCTCCT-3′, GAPDH forward: 5′-TATCGGACGCCTGGTTAC-3′, reverse: 5′-CTGTGCCGTTGAACTTGC-3′. In our prior work, we discussed the specifics of the PCR process and data analysis for the expression of Nrf2 utilizing the ABI Prism 7000 (Applied Biosystems, Carlsbad, CA, USA) by equation 2-ΔΔct.

Immunohistochemical Examination for NGF and Synaptophysin

Deparaffinization, rehydration, washing, immersion in percentage hydrogen peroxide, and digestion with pepsin for antigen retrieval were all performed on the tissue segment. The segment was treated with primary antibodies for NGF (monoclonal antibody, Cat # GTX03258, GeneTex, Inc, CA, USA, dilution 1: 200) and synaptophysin NGF (polyclonal antibody, Cat # GTX100865, GeneTex, Inc, CA, USA, dilution 1: 500) at 4°C overnight after serum had blocked non-specific binding. A brown signal was produced using the substrate diaminobenzidine and peroxidase enzyme. The section was counterstained, dehydrated, cleaned, then coverslipped. The main antibody is substituted with phosphate-buffered saline (PBS), and neighboring portions are utilized as a negative control. Using ImageJ software (National Institutes of Health, USA), the density of immunostaining was determined as the percentage of nerve area filled by positive staining (derived by averaging the data from ten fields at 10× magnification).

Statistical analysis

Data was processed and analyzed by GraphPad Prism software, Version 5.0 (GraphPad Software, Inc., San Diego, CA, USA). All data were expressed as mean ± SEM (standard error of the mean). A one-way ANOVA was used to find the statistical significance among all studied groups followed by Tukey's post hoc test to identify the significance between two separate groups. If p<0.05, it was considered significant.

## Results

Effects of antidiabetic agents on glycemic parameters in obese rats

Table 2 shows the results of the blood glucose, fasting insulin, HOMA index, total cholesterol, and triglycerides. There were significant rises in all of these parameters in the HFD group versus the control group (p<0.05). Also, serum glucose, insulin, cholesterol, and triglycerides showed significant attenuation in the metformin group, dapagliflozin, and semaglutide groups against the control group (p<0.05). Moreover, there has been no statistically significant difference among the metformin, dapagliflozin, and semaglutide groups.

**Table 2:** Effects of dapagliflozin and semiglutide on fasting blood glucose (mg/dl), fasting insulin (IU/dl), HOMA index, serum cholesterol (mg/dl), and triglycerides (mg/dl) in rats with HFD. All data were expressed as mean ± SEM; one-way ANOVA with Tukey’s post hoc test. * significant vs normal group; # significant vs. HFD group; $ significant vs metformin group; p<0.05 was considered significant. HOMA, homeostasis model assessment; HFD, high-fat diet; SEM, standard error of the mean.

	Normal group	HFD group	Metformin group	Dapagliflozin group	Semiglutide group
Blood glucose (mg/dl)	105.7± 4.45	144.4± 6.78^*^	99.95 ± 4.82^#^	120.0 ± 4.65^#^	124.7 ± 7.22^$^
Fasting insulin (IU/dl)	10.83 ± 1.14	16.83 ± 1.42^*^	9.33 ± 0.89^#^	10.5 ± 1.29^#^	10.67 ± 0.72^#^
HOMA index	2.82 ± 0.33	5.97 ± 0.55^*^	2.25 ± 0.21^#^	2.97 ± 0.32^#^	3.27 ± 0.25^#^
Serum cholesterol (mg/dl)	37.87 ± 2.35	75.17 ± 15.45^*^	51.84 ± 2.90	54.65 ± 6.57	53.85 ± 6.10
Serum triglycerides (mg/dl)	47.04 ± 2.54	122.1 ± 8.02^*^	61.23 ± 7.86^#^	62.51 ± 5.51^#^	68.17 ± 7.27^#^

Effects of antidiabetic agents on NCV studies in isolated sciatic nerve of obese rats

**Figure 1:**
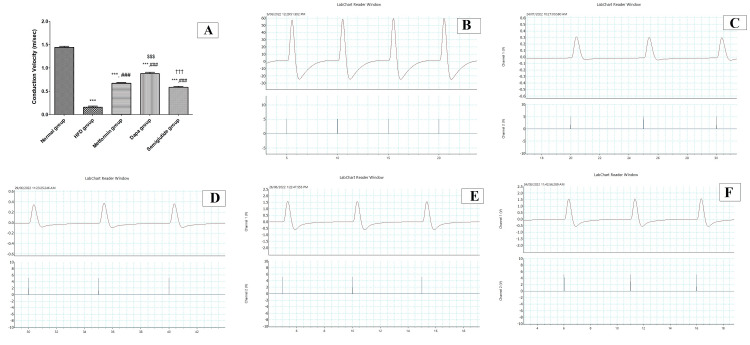
Nerve conduction study on isolated sciatic nerve. (A) NCV (m/sec) in isolated sciatic nerve in all experimental groups; (B) NCV traces normal group; (C) NCV traces in the HFD group; (D) NCV traces in the metformin group; (E) NCV traces in dapagliflozin (Dapa) group; and (F) NCV traces in semaglutide group. NCV, nerve conduction velocity; HFD, high-fat diet.

Figure 1A shows significant attenuation in NCV in isolated sciatic nerve in the HFD group in comparison to the control group (p<0.001). On the other hand, there were significant rises in NCV in metformin, dapagliflozin, and semaglutide groups compared to the HFD group (p<0.001). Moreover, there was a significant rise in NCV in the dapagliflozin group compared to metformin and semaglutide groups (p<0.001) and no statistically significant difference between metformin and semaglutide groups. Figures 1B-1F represent representative samples of NCV traces or records from the normal group, HFD group, metformin group, dapagliflozin, and semaglutide groups respectively.

Effects of antidiabetic agents on lipid peroxidation marker (MDA) and antioxidant genes (Nrf2 and HO-1) in obese rats

**Figure 2:**
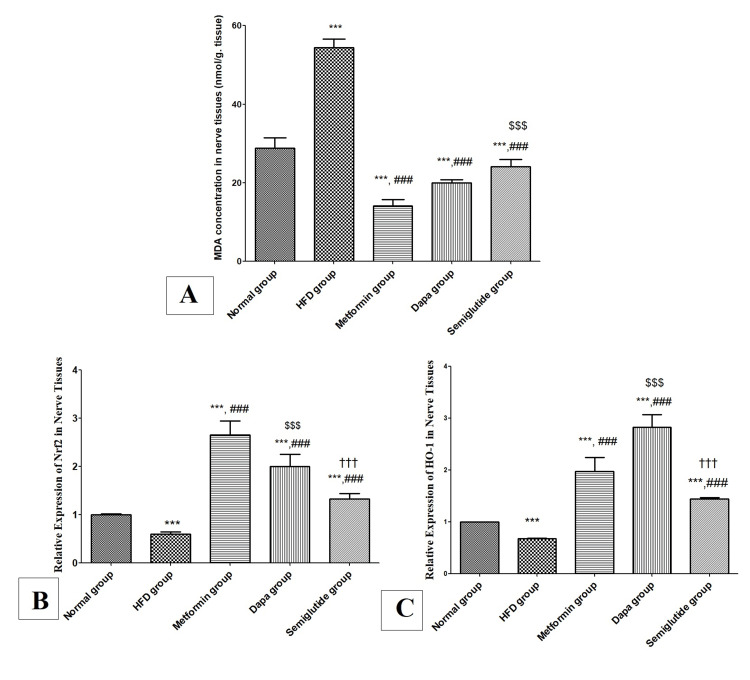
Oxidative stress markers and expression of antioxidant genes in the sciatic nerve. (A) MDA concentration (nmol/g nerve tissues) in nerve tissues in all studied groups; (B) relative expression of Nrf2 at mRNA level; and (C) relative expression of HO-1 at mRNA level in the sciatic nerve. Variables were expressed as mean±SD. *** p<0.001 significant vs normal group; ### p<0.001 significant vs HFD group; $$$ p<0.001 significant vs metformin group; ††† p<0.001 significant vs dapagliflozin (Dapa) group. MDA, malondialdehyde; HFD, high-fat diet.

Figure 2A shows a significant rise in MDA concentration in nerve tissues in the HFD group compared to that of the normal group (p<0.001). Then again, there was a significant decrease in MDA concentration in metformin, dapagliflozin, and semaglutide groups as opposed to the HFD group (p<0.001). Moreover, there was a significant rise in MDA concentration in the semaglutide group compared to the metformin group (p<0.001) and there is no statistically meaningful variance between metformin and dapagliflozin groups.

Figures 2B, 2C show the results of the relative expression of Nrf2 and HO-1 antioxidant genes in the sciatic nerve respectively. There was significant attenuation in the expression of Nrf2 and HO-1 genes at mRNA levels in nerve tissues in the HFD group compared to that of the normal group (p<0.001). In contrast, there was a significant increase in Nrf2 and HO-1 gene expression in metformin, dapagliflozin, and semaglutide groups in comparison to the HFD group (p<0.001). Besides, the dapagliflozin group showed a significant rise in HO-1 expression relative to the metformin group, while the semaglutide group showed a significant decrease in Nrf2 and HO-1 expression compared to metformin and dapagliflozin groups (p<0.001).

Effects of antidiabetic agents on lipid contents in sciatic nerve tissues in obese rats

**Figure 3:**
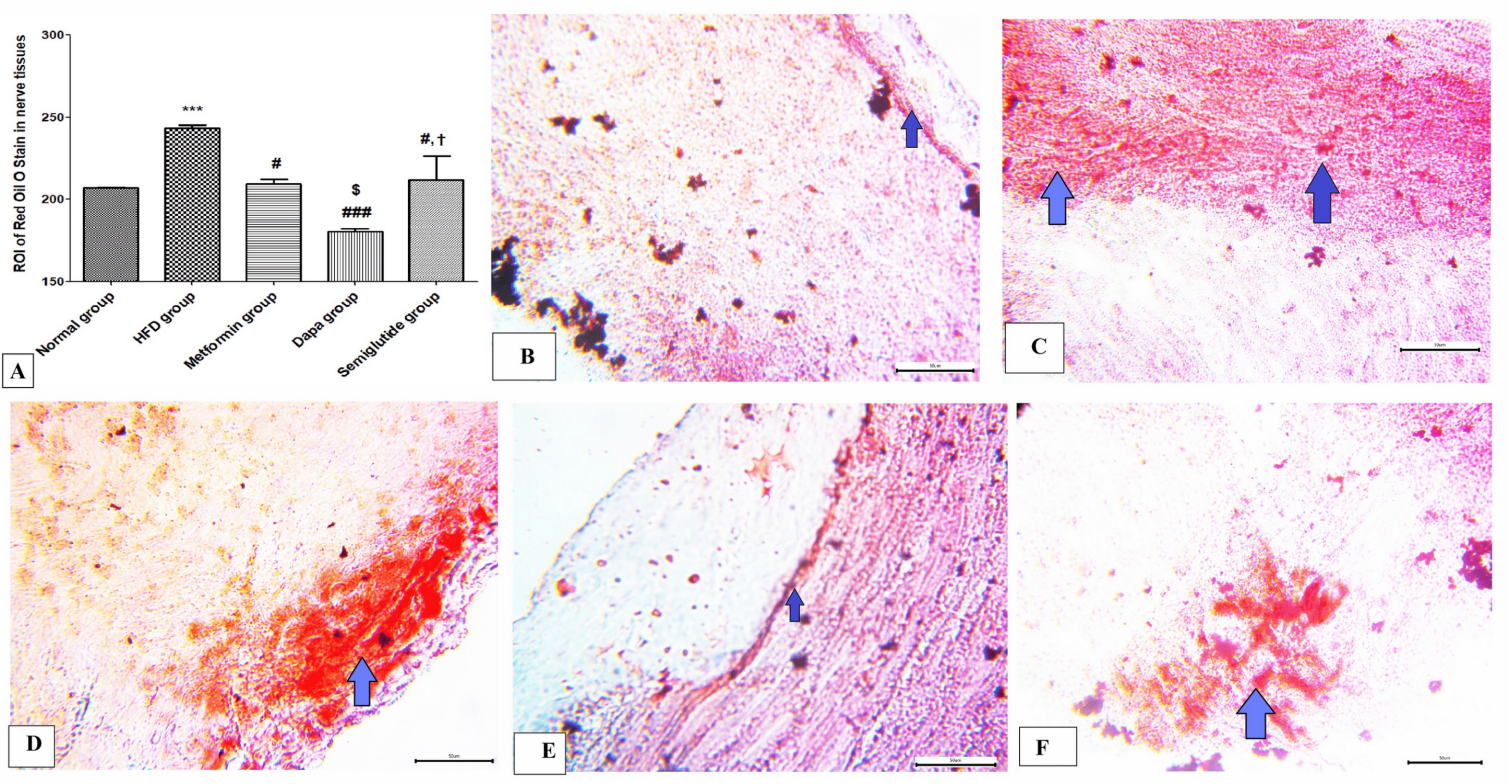
Oil red O staining for fat accumulation in the sciatic nerve. (A) Score of oil red O stain in the sciatic nerve in different studied groups; (B-F) representative samples of oil red O stain from normal, HFD, metformin, dapagliflozin (Dapa), and semaglutide groups respectively (lipid content stained by oil red O stain, blue arrows). Variables were expressed as mean±SD. *** p<0.001 significant vs normal group; ### p<0.001 significant vs HFD group; $$$ p<0.001 significant vs metformin group; and ††† p<0.001 significant vs dapagliflozin (Dapa) group. HFD, high-fat diet; ROI, region of interest.

Figure 3A shows the results of the oil red O stain (indicating the fat contents) in the sciatic nerve. There was a considerable increase in the density of the mean area of interest (ROI, i.e., region of interest) of oil red O stain in nerve tissues in the HFD group in comparison to the normal group (p<0.001). Then again, there was a significant reduction in oil red O stain in metformin, dapagliflozin, and semaglutide groups compared to the HFD group (p<0.05). Furthermore, the dapagliflozin group showed a large decrease in oil red O stain relative to the metformin and semaglutide groups (p<0.001). Figures 3B-3F are representative samples of oil red O stain from normal, HFD, metformin, dapagliflozin, and semaglutide groups respectively.

Effects of antidiabetic agents on morphology and ultrastructure of sciatic nerve tissues in obese rats

**Figure 4:**
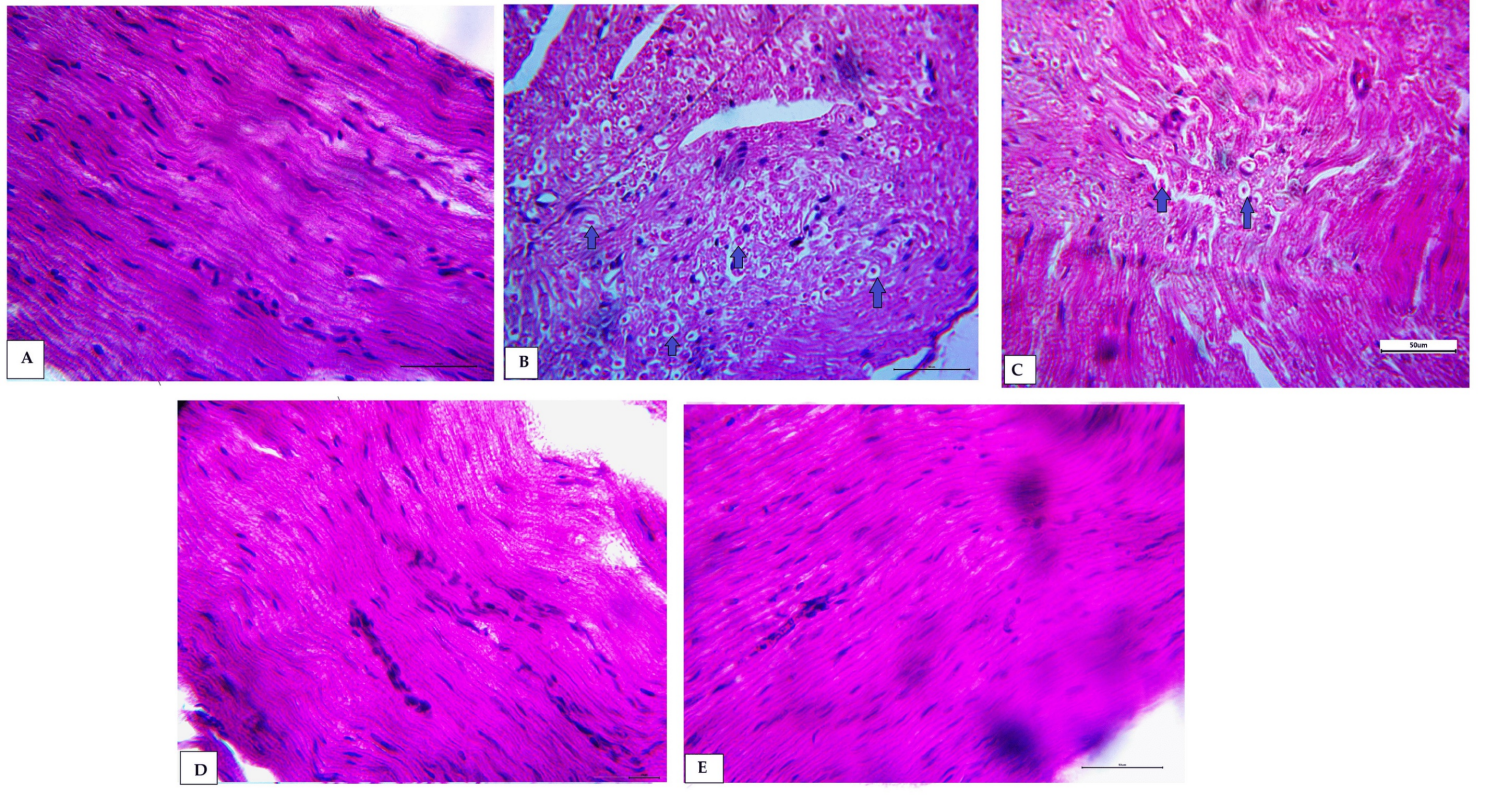
H&E staining for the sciatic nerve in all experimental groups. (A) Normal group (400x) shows normal and regular arrangement of nerve fibers; (B) HFD group (400x) shows irregular arrangement of nerve fibers with wide spacing and interstitial edema and perineural vacuolization (fatty depositions, blue arrows) (400x); (C) semaglutide group shows irregular arrangement of nerve fibers and interstitial edema (blue arrows) (400x); (D) metformin group shows nearly normal and regular arrangement of nerve fibers (400x); and (E) dapagliflozin group shows regular arrangement of nerve fibers (400x). H&E, hematoxylin and eosin; HFD, high-fat diet.

H&E staining for sciatic nerve demonstrated that the control group had consistently distributed myelinated fibers with normal shapes, undamaged myelin sheaths, and axon thickness proportionate to diameter (Figures 4A, 4B). On the other hand, sciatic nerve tissues from the HFD group showed sparsely distributed myelinated fibers with wide spacing between nerve fibers and myelin sheath deformation (Figure 4C). In treated groups, metformin and dapagliflozin groups showed regular and uniformly disturbed nerve fibers (Figures 4D, 4E), while the semaglutide group showed dispersed nerve fibers and wide spacing between nerve fibers (Figure 4F).

**Figure 5:**
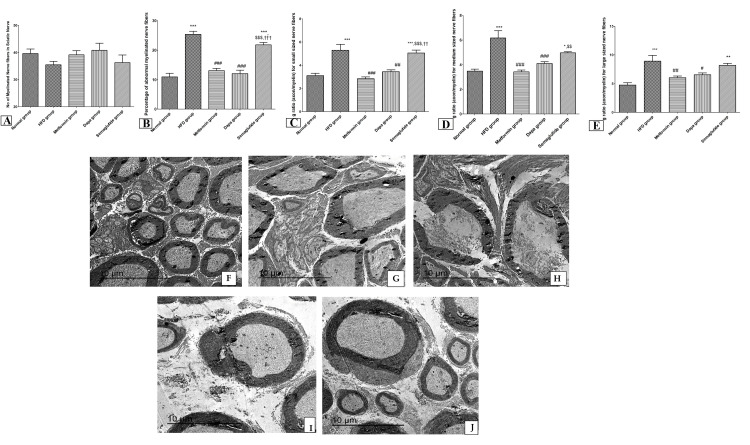
EM examination of the sciatic nerve in all experimental groups. (A) The number of myelinated nerve fibers in the sciatic nerve; (B) percentage of abnormal myelinated nerve fibers; (C) g-ratio for small-sized nerve fibers; (D) medium-sized nerve fibers; and (E) large-sized nerve fibers. Photomicrographs using a TEM for (F) normal group; (G) HFD group; (H) semaglutide group; (I) metformin group; and (J) dapagliflozin (Dapa) group, all in magnification 12000x. Variables were expressed as mean±SD. *** p<0.001 significant vs normal group; ### p<0.001 significant vs HFD; $$$ p<0.001 significant vs metformin; and ††† p<0.001 significant vs dapagliflozin (Dapa) group. EM, electron microscope; TEM, transmission electron microscope; HFD, high-fat diet.

Electron microscope (EM) examination revealed a non-significant decrease in the proportion of myelinated nerve fibers with a significant increase in the percentage of abnormal myelinated nerve fibers and an increase in g (axon/myelin)-ratio of small-, medium-, and large-sized nerve fibers in HFD group in comparison to the control group (p<0.001). Conversely, metformin and the dapagliflozin groups showed a significant decline in the percentage of abnormal myelinated nerve fibers and g-ratio for all sized nerve fibers (p<0.05). Likewise, the semaglutide group revealed a considerable rise in the abnormal myelinated nerve fibers and g-ratio for small- and medium-sized nerve fibers compared to metformin and dapagliflozin groups (p<0.05) (Figures 5A-5E). Figures 5F-5J are representative samples of EM from normal, HFD, metformin, dapagliflozin, and semaglutide groups respectively.

Effects of antidiabetic agents on NGF and synaptophysin expression in sciatic nerve tissues in obese rats

**Figure 6:**
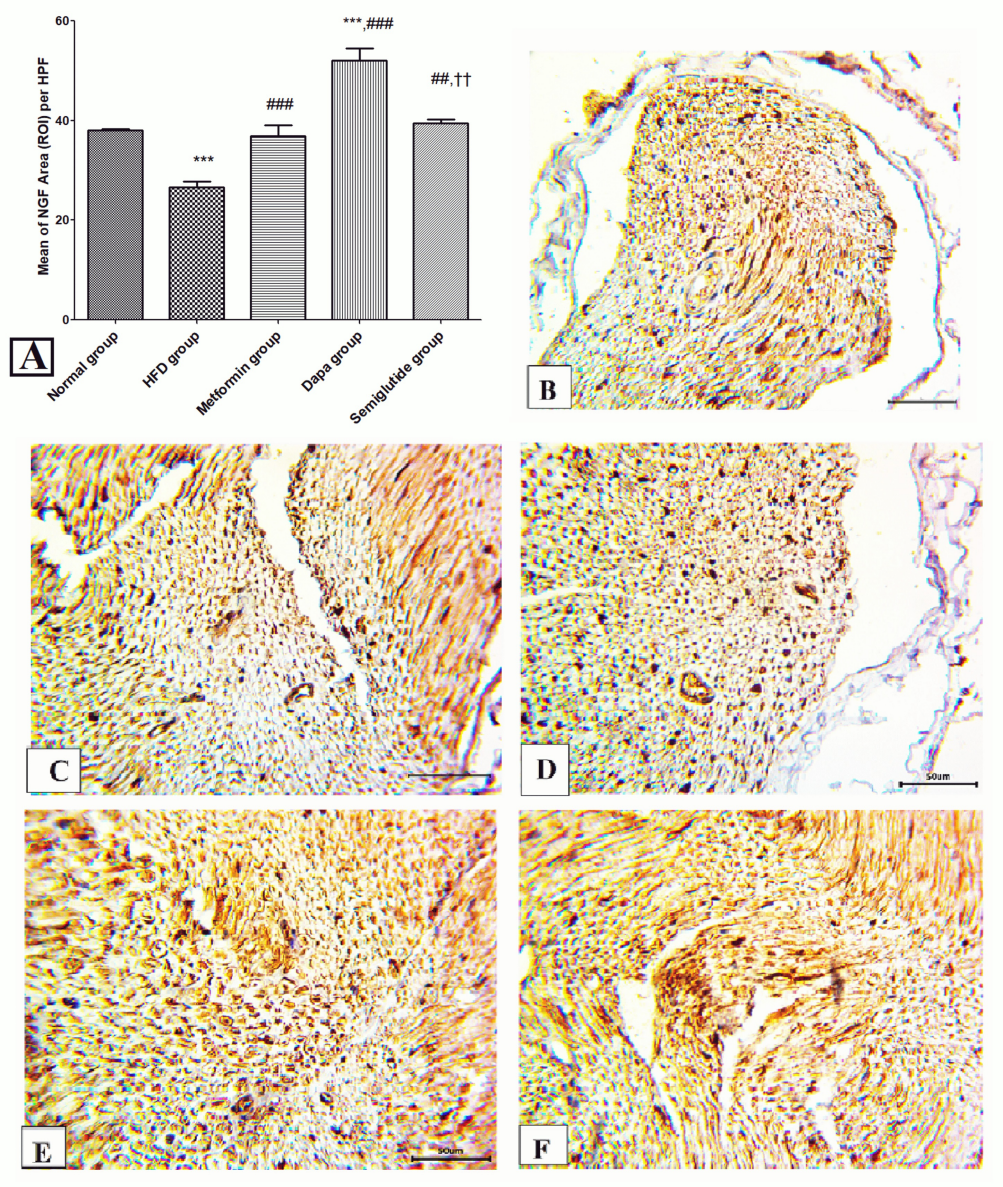
Expression of NGF in immunohistochemical results. (A) Mean of NGF area (ROI) per HPF; immunostaining for NGF shows brown cytoplasmic staining in (B) control group, (C) HFD group, (D) metformin group, (E) dapagliflozin (Dapa) group, and (F) semaglutide group. (B-F) All in magnification 400x. Variables were expressed as mean±SD. *** p<0.001 significant vs normal group; ### p<0.001 significant vs HFD, $$$ p<0.001 significant vs metformin, and ††† p<0.001 significant vs dapagliflozin (Dapa) group. NGF, nerve growth factor; ROI, region of interest; HPF, high-power field; HFD, high-fat diet.

Figure 6A shows the expression of NGF in immunohistochemical results. There was a significant attenuation in the area stained with NGF in the HFD group in comparison to the control group (p<0.001). On the contrary, there was a significant increase in NGF expression in metformin, dapagliflozin, and semaglutide groups as compared to the HFD group (p<0.05). Moreover, the dapagliflozin group exposed a meaningful rise in the NGF-stained area relative to metformin and semaglutide groups (p<0.001). Figures 6B-6F are representative samples of nerve specimens from normal, HFD, metformin, dapagliflozin, and semaglutide groups respectively.

**Figure 7:**
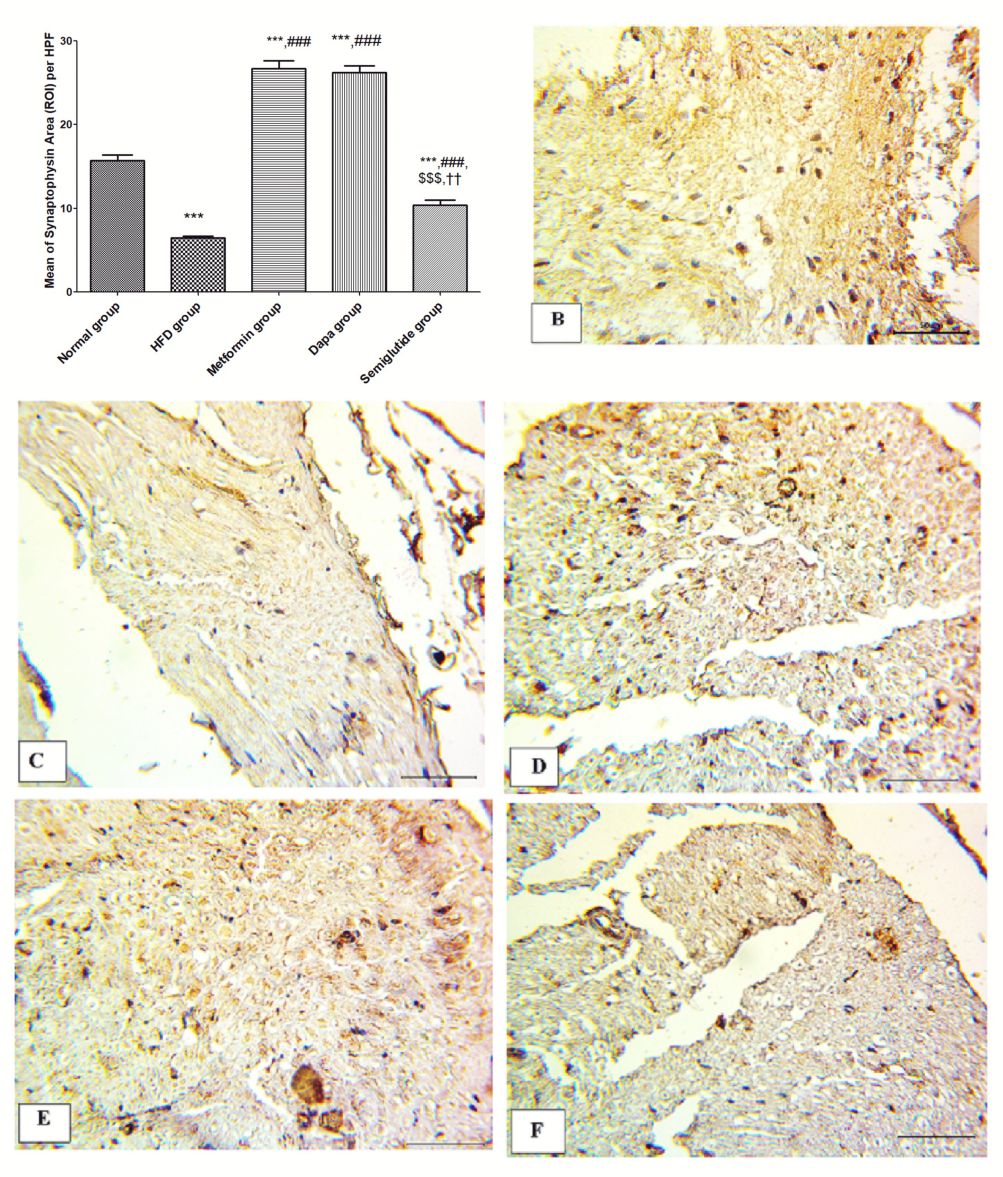
Expression of synaptophysin in immunohistochemical results. (A) Mean of synaptophysin area (ROI) per HPF; immunostaining for synaptophysin shows brown cytoplasmic staining in (B) control group, (C) HFD group, (D) metformin group, (E) dapagliflozin (Dapa) group, and (F) semaglutide group. (B-F) All in magnification 400x. Variables were expressed as mean±SD. *** p<0.001 significant vs normal group, ### p<0.001 significant vs HFD, $$$ p<0.001 significant vs metformin, and ††† p<0.001 significant vs dapagliflozin (Dapa) group. ROI, region of interest; HPF, high-power field; HFD, high-fat diet.

Figure 7A shows the expression of synaptophysin in immunohistochemical results. There was a significant attenuation in the area stained with synaptophysin in the HFD group compared to that of the control group (p<0.001). Besides, there was a significant increase of synaptophysin expression in metformin, dapagliflozin, and semaglutide groups as compared to the HFD group (p<0.05). Noted that the dapagliflozin group showed a significant increase in synaptophysin stained area relative to metformin and semaglutide groups (p<0.001). Figures 7B-7F are representative samples of nerve specimens from normal, HFD, metformin, dapagliflozin, and semaglutide groups respectively.

## Discussion

The most significant avoidable cause of mortality in the 21st century is obesity, which is a very serious health issue that is spreading like an epidemic all over the world. Since 1980, the number of obese persons has increased globally, according to statistics from the WHO [1]. Consequently, the goal of the study was to compare fat accumulation and nerve conduction velocity evaluation in obese Sprague Dawley rats and those that received different new anti-hyperglycemic drugs; SGLT2 inhibitor and GLP1 analog.

In the current research study, HFD groups revealed a noteworthy rise in blood glucose, fasting insulin, HOMA index, triglycerides, and total cholesterol. These findings agree with Ahangarpour et al. and suggest impairment of lipid and glucose metabolism and development of IR in obese rats [14]. Interestingly, the metformin, dapagliflozin, and semaglutide groups showed significant attenuation in these parameters [11]. Antihyperglycemic drugs achieved these changes by decreasing the hepatic gluconeogenesis, significantly able to increase serum insulin level probably by enhancing expression and function of GLUT4 in cells and by stimulating GLP1 release, both effects enhance the secretion of insulin from pancreatic bets cells. In addition, these drugs affect the lipoprotein synthesis in the intestine of diabetic rats by the reduction of mRNA expression of genes responsible for intestinal lipid homeostasis [15].

The present work reported that the HFD group showed significant attenuation in NCV in isolated sciatic nerve, in line with Coppey et al. who stated that diabetes mellitus causes pronounced abnormalities in nerve conduction, possibly as a result of impaired vasodilator function in the arterioles that supply blood to the sciatic nerve region [16]. This is linked to decreased endoneural blood flow (EBF), and because these defects occur before motor NCV slows, they may be a factor in nerve dysfunction [16]. These findings suggest impairment of the NCV in obese rats. On the other hand, there were significant rises in NCV in metformin, dapagliflozin, and semaglutide groups suggesting neuroprotective action for these agents in obese rats. Moreover, there was a significant rise in NCV in the dapagliflozin group compared to metformin and semaglutide groups. In line with the results of NCV, the nerve structure by histopathological routine examination and electron microscope showed similar findings. Histological examination revealed deteriorations of the nerve morphology and nerve fiber arrangements and g-ratio (index for the degree of myelination of nerve fibers) in all sized nerve fibers suggesting degeneration of myelin sheaths in the HFD group and explaining the marked reduction in nerve conduction velocity in obese rats in agreement with Bouhrara et al. [17]. On the other hand, treated groups (metformin, dapagliflozin, and semaglutide) showed significant improvement in g-ratio and degree of myelination in nerve fibers explaining the improvement of NCV in these groups [17]. These results correspond to Esmaeilnejad et al., who reported the buffering impact of metformin on myelin degeneration in obesity [18].

The amount of fat deposition in nerve tissues was assessed by oil red O stain. In the current study, the HFD group revealed a considerable rise in the density of mean area of interest (ROI) of oil red O stain in nerve tissues indicating excessive fat accumulation in nerve tissues of rats received HFD. The results of this study are in line with those stated by Baek et al. [19]. On the other hand, there was a significant reduction in oil red O stain in metformin, dapagliflozin, and semaglutide groups suggesting their lipid-lowering effect, especially the dapagliflozin group which demonstrated marked attenuation in oil red O staining [19]. This result is consistent with a previous study, that demonstrated that dapagliflozin can protect against steatosis through the inhibition of NLRP3 inflammasome resulting in the reduction of high triglycerides, free fatty acids, and blood sugar in HFD [20].

Oxidative stress and inflammation play critical roles in the pathogenesis of obesity and its complications. As a result, in the current investigation, we evaluated the oxidative stress state in nerve tissues. In the current work, the HFD group revealed a noteworthy elevation in MDA concentration (a marker of lipid peroxidation) in nerve tissues along the lines of Nour et al., who reported that obesity may trigger oxidative stress and low-grade chronic inflammation [21]. However, there was a huge decline in MDA concentration in metformin, dapagliflozin, and semaglutide groups [21]. Noted that MDA level attenuated significantly in the metformin group in agreement with Naghdi et al., who stated that metformin has an antioxidant effect, and thereby might be therapeutically beneficial in improving diseases with oxidative stress basis in their pathogenesis [22]. Zaibi et al. stated that dapagliflozin has a beneficial effect in protecting against oxidative stress [23]. In addition, Liu et al. reported that semaglutide can inhibit oxidative stress [24]. 

 Concerning the antioxidant genes, there was significant attenuation in the expression of the transcription factor Nrf2 gene and its target HO-1 gene at mRNA levels in nerve tissues in HFD. These findings are similar to those reported by Kim et al. and Lestari et al. [25,26]. However, there was a huge rise in Nrf2 and HO-1 gene expression in metformin, dapagliflozin, and semaglutide groups, which may be due to their antioxidant activity. Moreover, the dapagliflozin group showed a significant rise in HO-1 expression relative to the metformin group in agreement with Refaie et al., who stated that dapagliflozin may achieve its protective effect through rising levels of glutathione (GSH) and glutathione peroxidase (GPx) in tissues along with the total antioxidant capacity (TAC) [27]. Noteworthy, the semaglutide group revealed a considerable decline in Nrf2 and HO-1 expression compared to metformin and dapagliflozin groups suggesting that semaglutide has less antioxidant activity than other antidiabetic drugs [27].

Concerning the immunohistochemical results, there was a significant reduction in the area stained with either NGF or synaptophysin in the HFD group in accordance with Korkmaz et al., who stated that the expression of synaptophysin has been decreased in HFD [28]. That explains why obesity leads to defective synaptic plasticity and, therefore, deterioration in cognitive function. On the contrary, there was a significant increase in NGF and synaptophysin expression in metformin, dapagliflozin, and semaglutide groups, and, in particular, the dapagliflozin group. Suggesting that the antidiabetic drugs have a protective effect in obesity-induced neuropathic changes, specifically, the dapagliflozin group.

Strengths and limitations

The current study is the first study, to the best of our knowledge, to demonstrate the neuroprotective effects of GLP1 analog and SGLT2 inhibitors against obesity-induced peripheral neuropathy in rats. Although the current study tried to examine the mechanisms underlying the protective actions of these agents and found the role of oxidative stress, NGF, and synaptophysin, further studies are needed for full elucidation of the exact mechanisms of obesity-induced such as autophagy, apoptosis, and inflammatory cytokines.

## Conclusions

Along with regulating the glycemic state in obese rats, new anti-diabetic medications like SGLT2 inhibitors and GLP1 analogs may also offer neuroprotective advantages in rat models of multiple sclerosis. This outcome may result from decreased oxidative stress and increased levels of Nrf2, HO-1, synaptophysin, and nerve growth factor in the neural tissues of obese rats. More neuroprotective effects were provided by SGLT2 inhibitors than by GLP1 analogs.
